# Wolff-Parkinson-White syndrome: where is the pathway?

**Published:** 2009-03-15

**Authors:** Mintu P Turakhia, Melvin Scheinman, Nitish Badhwar

**Affiliations:** 1 Electrophysiology Section, Department of Medicine, Stanford University;; 2 Veterans Affairs Palo Alto Health Care System; 3 Cardiac Electrophysiology Section, Department of Medicine, University of California, San Francisco

**Keywords:** Wolff-Parkinson-White, pre-excitation, accessory pathway, ablation, coronary sinus ablation

## Abstract

A 31-year old male presented with atrial fibrillation and ventricular preexcitation that was positive in leads V1-V4, negative in lead II, and positive in lead AVR. The patient was cardioverted and invasive electrophysiologic study was performed. Based on the ECG findings, the coronary sinus and its branches were interrogated during orthodromic atrioventricular reentrant tachycardia. The earliest local activation was seen in the true coronary sinus lumen at the bifurcation of the posterolateral branch. Radiofrequency energy application at this area led to loss of preexcitation. When localizing left septal and posterior accessory pathways, ventricular preexcitation that is both negative in II and positive in AVR has been shown in previous studies to be highly sensitive and specific for a subepicardial location. Therefore, investigation of the coronary sinus and its branches may allow for effective ablation without the need for left ventricular access.

## Case Presentation

**Figure 1:**
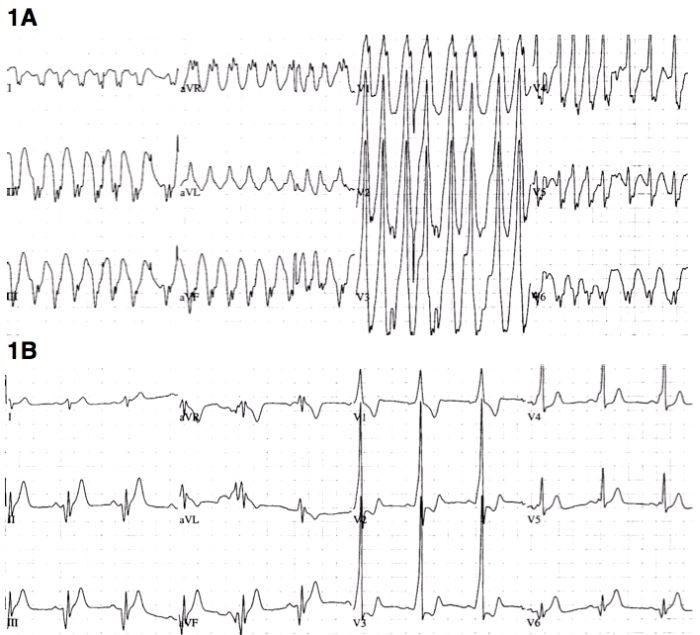
Electrocardiogram before (1A) and after (1B) electrical cardioversion

A 31-year old male presented to the emergency room with palpitations. The electrocardiogram (ECG) showed atrial fibrillation with preexcitation (Figure 1A). After electrical cardioversion, there was evidence of preexcitation in sinus rhythm (Figure 1B). Invasive electrophysiology study was performed, which confirmed the presence of a single accessory pathway. What is the most likely site of the pathway, and from where should you start to map?

## Discussion

Figure 1A demonstrates a wide complex tachycardia with varying R-R intervals and QRS duration, consistent with atrial fibrillation with varying degrees of preexcitation. In figure 1B, there is sinus rhythm with a short PR interval and delta waves that are positive in leads V1-V4, a finding that is highly specific for left-sided accessory pathways [1,2]. The initial forces have a superior axis (positive in AVR, negative in II, III, AVF) with activation toward the lateral leads (I and AVL).

The surface ECG during atrial fibrillation also allows for examination of purely preexcited beats. The QRS is still positive in leads V1-V3, consistent with a left sided accessory pathway. Leads II, III, and AVF are negative, consistent with a posterior location.

At this point, left-sided access could be considered for mapping and ablation in the left paraseptal or mitral annular region. However, there are still additional clues to further localize the pathway. Most notably, AVR is positive with preexcitation (Figure 1), which is highly specific for a subepicardial focus [3,4]. In two case series of septal and paraseptal pathways, AVR positivity had a specificity of 99% [3] and 100% [4] for subepicardial localization and successful ablation from within the coronary sinus. In subepicardial pathways, a negative QRS in lead II is almost always present (86-100%), but nonspecific, as this may be seen in other posterior septal and paraseptal locations.

**Figure 2:**
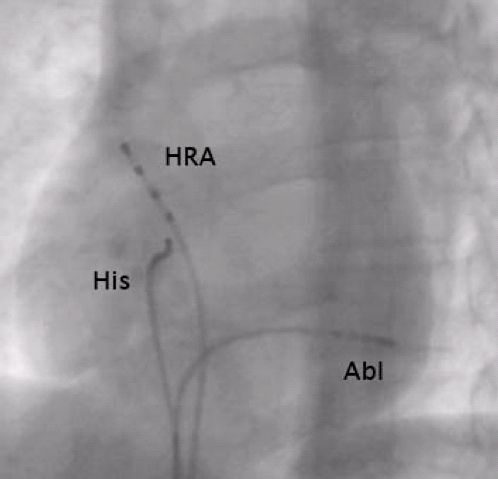
Left anterior oblique fluoroscopic image from electrophysiology study showing the ablator position at the successful site in the coronary sinus. HRA = high right atrial catheterHis = bundle of His catheterAbl = ablation catheter

**Figure 3:**
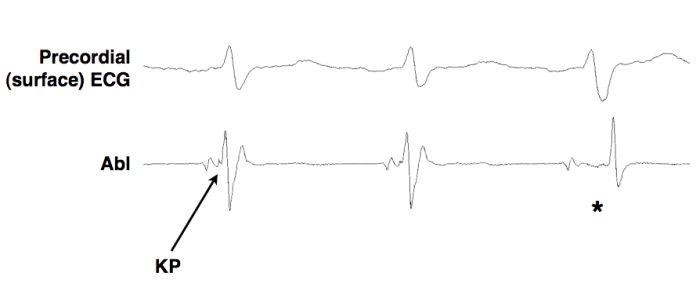
Electrogram in sinus rhythm during application of radiofrequency energy. Kent potential (KP) on ablation catheter (Abl) disappears (*) and there is abrupt local A-V interval prolongation and a subtle change in the surface QRS, indicating loss of preexcitation. Abl = ablation catheter KP = Kent potential

Based on these ECG criteria, the coronary sinus and its branches were initially interrogated during orthodromic atrioventricular reentrant tachycardia. The earliest local activation was seen in the true coronary sinus lumen at the bifurcation of the posterolateral branch (Figure 2). At this area, an initial early sharp deflection was observed, consistent with a Kent potential (Figure 3).

Radiofrequency ablation was performed in sinus rhythm at this site with a 4 mm ablation catheter and low energy settings. (power 20W; temperature limit 50ºC). After 6.3 seconds of RF during sinus tachycardia, there was an abrupt increase in the local A-V interval, loss of the Kent potential, and loss of preexcitation (Figure 3). After a 30-minute waiting period, there was no evidence of preexcitation in the baseline state, with atrial pacing or induced atrial fibrillation.

In this case, trans-septal puncture or retrograde arterial access to map the left ventricular endocardium was avoided. When localizing left septal and posterior accessory pathways, ventricular preexcitation that is both negative in II and positive in AVR has been shown in previous studies to be highly sensitive and specific for a subepicardial location. Up to eight percent of septal and paraseptal pathways may be epicardial [3-5]. Therefore, investigation of the coronary sinus and its branches may allow for effective ablation without the need for left ventricular access.
